# Mixed reality guidance improves humeral head cut accuracy

**DOI:** 10.1016/j.jsea.2026.100105

**Published:** 2026-09-30

**Authors:** Daniel B. Calem, Lorik Berisha, Tej Joshi, Akhil Katakam, John Erickson

**Affiliations:** a Department of Orthopaedic Surgery, Rutgers New Jersey Medical School, Newark, NJ, USA; b Department of Orthopaedic Surgery, New York University, New York, NY, USA; c Overlook Medical Center, Orthopaedic Surgery, New Providence, NJ, USA

**Keywords:** Mixed reality, Holographic guidance, Shoulder arthroplasty, Humeral head osteotomy, Surgical navigation, Augmented reality

## Abstract

**Background:**

The humeral head osteotomy is a critical step in both anatomic and reverse shoulder arthroplasty because it determines humeral component alignment, including neck-shaft angle (NSA), retroversion, and component height. Inaccurate osteotomy may adversely affect implant positioning, soft tissue tensioning, joint biomechanics, and long-term clinical outcomes. Mixed reality (MR) technology has demonstrated potential to improve intraoperative visualization and surgical accuracy in shoulder arthroplasty; however, its role in improving humeral head osteotomy accuracy remains largely unexplored. The purpose of this study was to determine whether MR guidance improves the accuracy of humeral head osteotomy compared with freehand and extramedullary guide (EG) techniques.

**Methods:**

Forty-five identical 3-dimensional printed arthritic proximal humerus models were created and mounted in standardized orientation. Nine orthopedic surgery residents were stratified by postgraduate year and randomly assigned within each stratum to one of 3 groups performing humeral head osteotomies using one of 3 techniques: freehand, EG, or MR holographic guidance using a Microsoft HoloLens 2. Each participant performed 5 osteotomies targeting a 135° NSA and 30° of retroversion. Following osteotomy, specimens were digitized using 3-dimensional scanning and compared with a predefined reference osteotomy plane using iterative closest point analysis in Blender software. Primary outcomes included absolute deviation from planned NSA, retroversion, and cut height.

**Results:**

Forty-two osteotomies were included in the final analysis, including 15 MR-assisted osteotomies, 15 freehand osteotomies, and 12 EG osteotomies. MR-assisted osteotomy demonstrated significantly lower absolute NSA deviation compared with both freehand and EG techniques. Mean absolute NSA deviation was 5.6° ± 5.2° in the MR group, compared with 10.4° ± 6.3° in the freehand group and 10.4° ± 5.2° in the EG group (*P* = .013). MR guidance also demonstrated significantly lower absolute cut height deviation (4.2 ± 2.0 mm) compared with freehand (7.8 ± 3.0 mm) and EG techniques (7.1 ± 3.5 mm) (*P* = .008). No significant differences in retroversion deviation were identified between groups (*P* = .70).

**Conclusion:**

MR holographic guidance improved the accuracy and reproducibility of humeral head osteotomy, particularly with respect to NSA and cut height, compared with freehand and EG techniques. These improvements were achieved using passive holographic guidance without formal navigation, optical tracking, or hologram registration, suggesting that 3-dimensional visualization alone may improve osteotomy reproducibility during shoulder arthroplasty. Further investigation is warranted in cadaveric and clinical settings.

The humeral head osteotomy determines humeral component alignment in both anatomic and reverse shoulder arthroplasty. The cut sets the neck-shaft angle (NSA), retroversion, and component height. In anatomic shoulder arthroplasty, restoration of native inclination and version reproduces normal glenohumeral kinematics, preserves rotator cuff tension, and limits eccentric glenoid loading.^9^^,^^13^^,^^27^^,^^31^ In reverse shoulder arthroplasty, these same 3 variables govern deltoid tensioning, prosthetic stability, scapular notching, arm lengthening, and acromial stress.^2^, ^14^, ^15^, ^17^, ^18^, ^24^

Reproducing a planned osteotomy is technically difficult. Native NSA ranges from approximately 130° to 140°, and the humeral version varies substantially between patients.^9^^,^^21^^,^^31^ The proximal humerus is exposed through a limited window, osteophytes obscure the anatomic neck in the arthritic shoulder, and version is judged against the forearm axis rather than against a fixed landmark within the surgical field. Freehand osteotomy has demonstrated substantial variability in inclination, version, and cut height.^12^, ^29^, ^30^

Several technologies address this problem. Extramedullary guides (EGs) reference the forearm axis mechanically and remain the most widely available adjunct. Patient-specific instrumentation, computer navigation, and computed tomography-based 3-dimensional(3D) planning improve implant positioning accuracy but add cost, pre-operative planning time, workflow complexity, and dependence on advanced imaging.^1^, ^4^, ^5^, ^11^, ^19^, ^22^, ^26^, ^32^

Mixed reality (MR) differs from virtual reality, which immerses the user in a fully digital environment, and from augmented reality, which overlays static digital content onto the real world. MR allows the user to manipulate virtual 3-dimensional objects while remaining anchored within the physical operative field.^3^^,^^20^ An MR guide requires no additional incision-side hardware and no patient-specific manufacturing, and it presents the planned cut in 3 dimensions at the site of the osteotomy. In shoulder arthroplasty, MR has been applied almost exclusively to glenoid guide pin placement.^6^^,^^7^^,^^10^^,^^23^^,^^25^^,^^28^ Its application to the humeral side remains largely unexplored.

The purpose of this study was to determine whether MR guidance improves the accuracy of humeral head osteotomy compared with freehand and EG techniques. We hypothesized that MR-assisted osteotomy would demonstrate lower absolute deviation from the planned NSA, retroversion, and cut height than either freehand or EG osteotomy.

## Materials and methods

A total of 45 Sawbones models of arthritic proximal humeri were created using 3D-printed acrylonitrile butadiene styrene filament with 30% infill. Each model was an identical reproduction of the same arthritic proximal humerus, including identical osteophyte morphology (Fig. 1). A single proximal humerus model was intentionally utilized to standardize anatomic morphology across all osteotomy attempts and minimize confounding variables related to differences in proximal humeral anatomy, osteophyte formation, inclination, and version. All models were mounted in identical orientation throughout testing.

**Figure 1:**
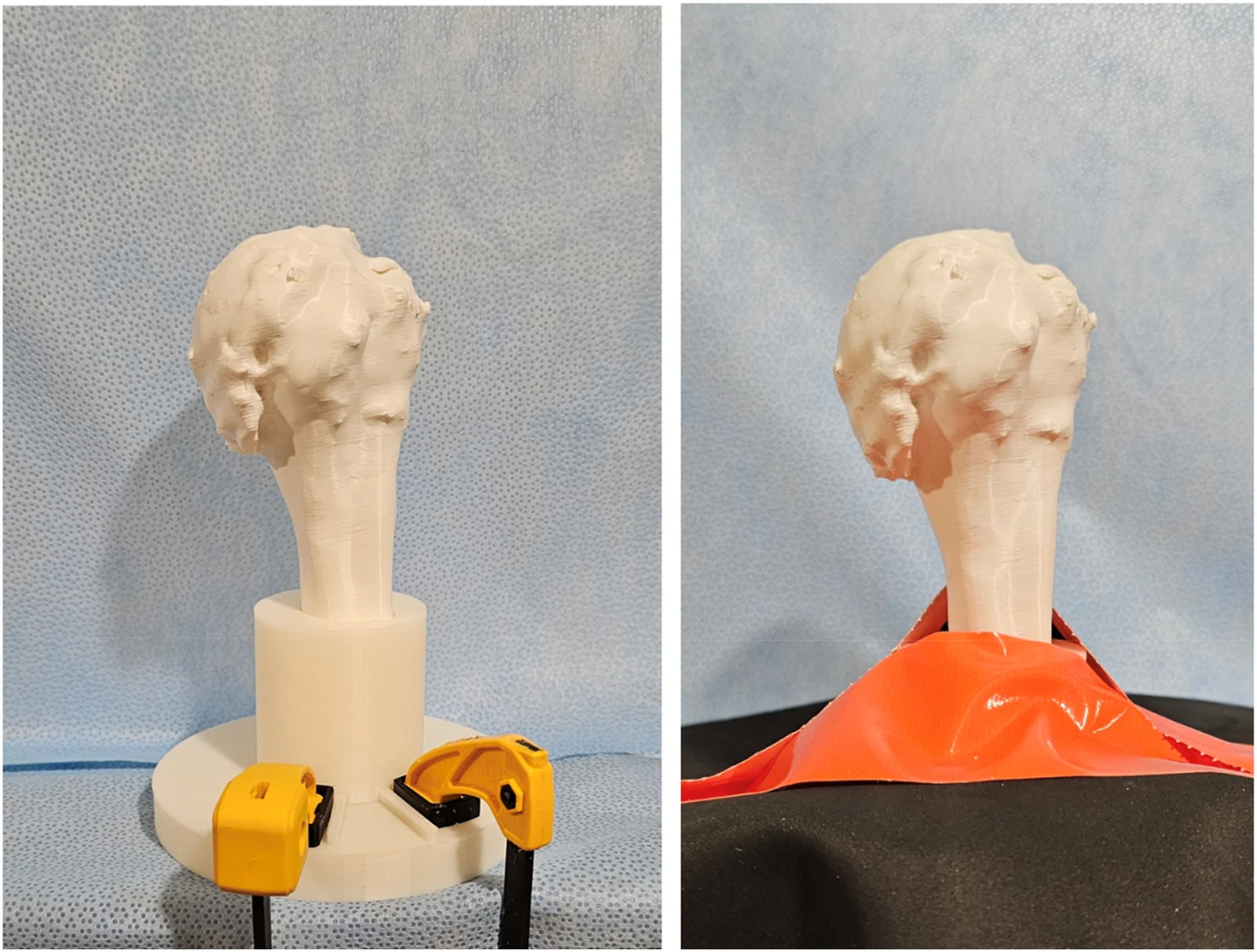
Standardized three-dimensional printed arthritic proximal humerus model and mounting apparatus. Identical three-dimensional printed arthritic proximal humerus models utilized for all osteotomy attempts. Models were rigidly fixed in standardized orientation using a custom mounting apparatus. The distal portion of the mount functioned as a surrogate forearm axis for retroversion referencing during osteotomy.

The models were rigidly mounted to the operating table using a custom fixation apparatus. While only the proximal portion of the mount is visible in Fig. 1, the distal portion extended inferiorly and functioned as a surrogate for the native elbow and forearm axis commonly referenced during shoulder arthroplasty. A full-length humerus model was intentionally not utilized because the humeral shaft and distal humerus are not fully visualized during standard shoulder arthroplasty exposure, and the goal was to simulate the limited operative exposure and intraoperative environment encountered clinically.

Three humeral head osteotomy techniques were evaluated: (1) freehand osteotomy without standard instrumentation, (2) osteotomy using a 135° EG, and (3) MR-assisted osteotomy using a Microsoft HoloLens 2 (Redmond, WA, USA). A single digitally templated osteotomy plane targeting a 135° NSA and 30° of retroversion was created pre-operatively and served as the reference model for all comparisons (Fig. 2).

**Figure 2:**
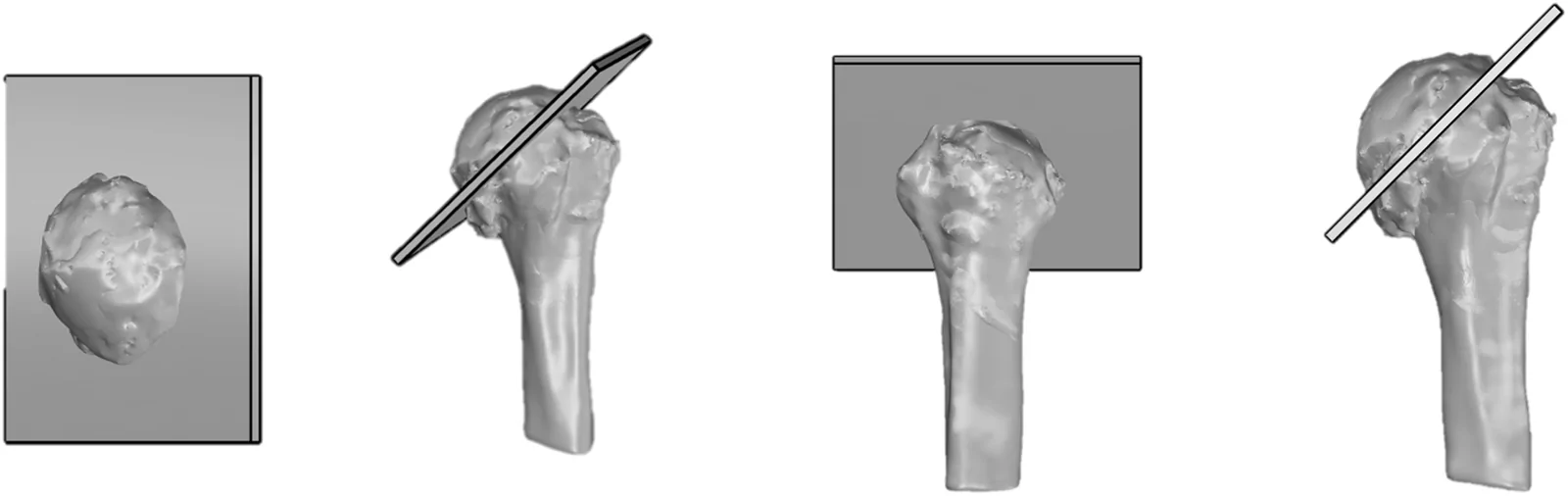
Pre-operative planning reference model demonstrating target osteotomy parameters. Digitally templated humeral head osteotomy reference model demonstrating the planned 135° neck-shaft angle and 30° of retroversion utilized for all osteotomy attempts and software-based comparisons.

Freehand osteotomy was performed without a cutting guide, jig, alignment rod, or any other instrumentation. Participants established the osteotomy plane visually from the surface anatomy of the model and cut directly with the oscillating saw.

EG osteotomy was performed using a 135° extramedullary cutting guide that was designed and 3-dimensionally printed by the senior author for this study rather than drawn from a commercial shoulder arthroplasty instrument set. Participants positioned the guide against the proximal humerus and used it as a physical reference for the intended cut plane. The version was set by aligning the version rod of the guide with the clamp securing the model to the table, which served as the surrogate for the forearm axis. No mark was made on any proximal humerus model in any group, and the osteotomy was performed with the same oscillating saw in all 3 groups. The guide therefore established the target plane but did not constrain the saw.

The MR group utilized a Unity-based MR application installed on a head-mounted display, which projected a manipulatable 3-dimensional hologram of the planned humeral head osteotomy. Participants were able to independently manipulate the hologram's position, orientation, and scale during each osteotomy attempt (Fig. 3). The hologram was not formally registered or linked to the physical Sawbones model and instead functioned as an interactive 3-dimensional visual reference during osteotomy. No optical tracking, instrument tracking, or formal navigation was utilized.

**Figure 3:**
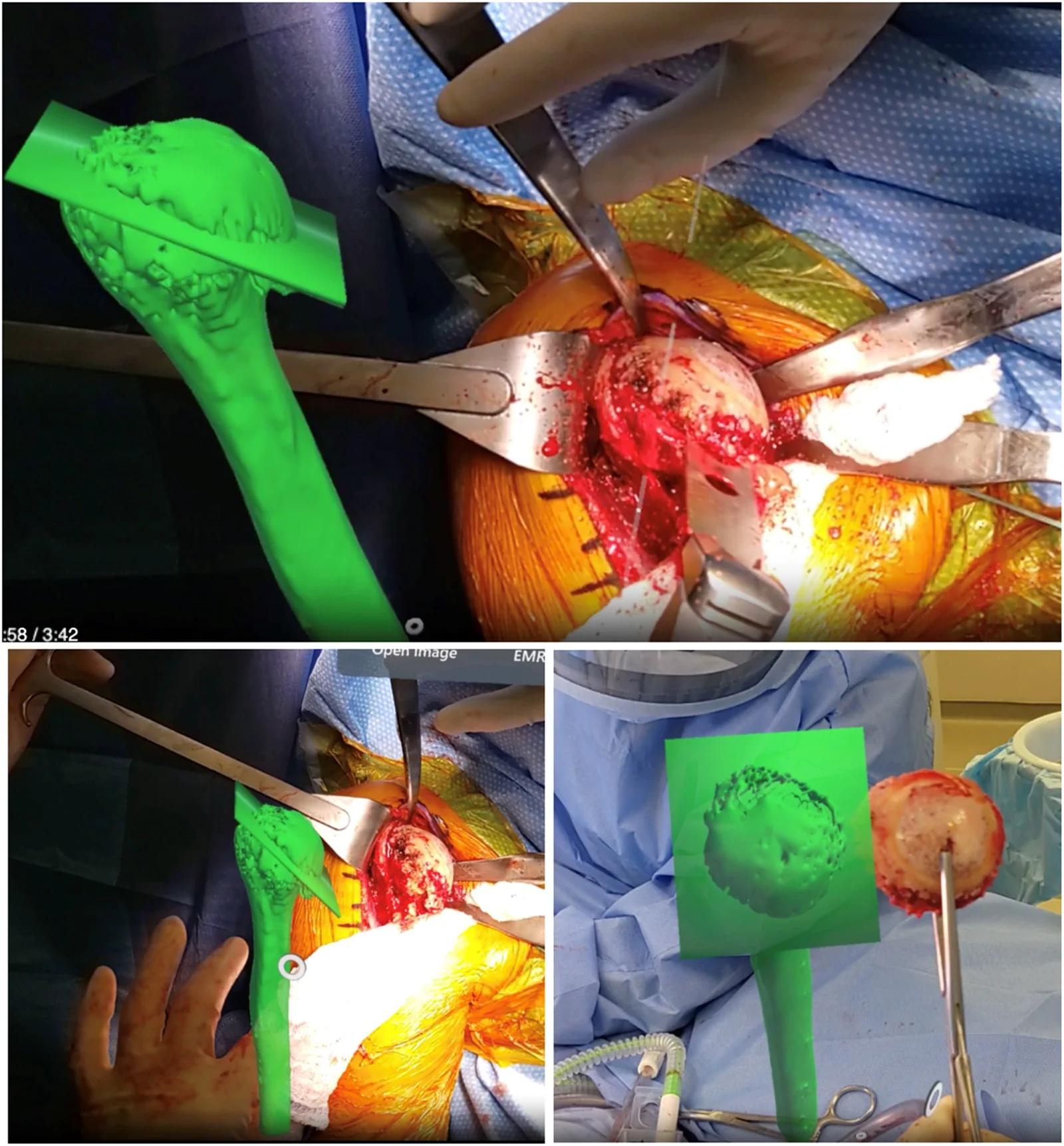
Mixed reality holographic guidance system. Surgeon point-of-view demonstrating the mixed reality holographic reference model displayed through the Microsoft HoloLens 2 head-mounted display during humeral head osteotomy. The hologram functioned as an interactive three-dimensional visual reference and was manipulatable in space and size by the participant.

Nine orthopedic surgery residents participated. Participants were stratified by postgraduate year (PGY) and randomly assigned within each stratum to one of the 3 technique groups (Table II). The MR group comprised one PGY-3, one PGY-4, and one PGY-5 resident; the EG group one PGY-2, one PGY-4, and one PGY-5 resident; and the freehand group 2 PGY-3 residents and one PGY-5 resident.

**Table I:** Mean absolute deviation from planned osteotomy parameters by technique.

Outcome	Mixed reality (n = 15)	Freehand (n = 15)	Extramedullary guide (n = 12)	*P* value
Neck-shaft angle deviation (°)	5.6 ± 5.2	10.4 ± 6.3	10.4 ± 5.2	.013
Retroversion deviation (°)	5.0 ± 3.3	5.4 ± 3.9	5.5 ± 3.1	.70
Cut height deviation (mm)	4.2 ± 2.0	7.8 ± 3.0	7.1 ± 3.5	.008

Mean absolute deviation from the planned neck-shaft angle (NSA), retroversion, and cut height for mixed reality (MR), freehand, and extramedullary guide (EG) osteotomy techniques. Values are reported as mean ± standard deviation. *P* values were calculated using linear mixed-effects models accounting for repeated measurements within participants.

All 9 participants had operated with a shoulder and elbow or sports medicine attending before the study. Prior experience performing a humeral head osteotomy during a case ranged from 0 to 10 osteotomies. That experience correlated more with interest in shoulder arthroplasty than training year; a PGY-3 intending to pursue shoulder and elbow surgery may have performed more proximal humeral osteotomies than a PGY-5 intending to pursue hip and knee arthroplasty. Stratification by PGY therefore balanced training level across the 3 groups but did not on its own equalize osteotomy-specific experience.

Residents rather than experienced shoulder arthroplasty surgeons were studied for 3 reasons. First, high-volume arthroplasty surgeons would be expected to perform near a ceiling on a standardized model, which would compress differences between techniques and obscure any effect attributable to guidance. Second, most shoulder arthroplasty in the United States is performed by surgeons with low annual case volume, and a resident cohort approximates the spatial referencing challenge faced by an intermittent operator. Third, if MR guidance reduces dependence on accumulated operative experience, that effect should be demonstrable first in less experienced operators.

No practice osteotomies were performed before data collection, and participants were not permitted to review the results of their own osteotomies at any point during testing. Each participant received a standardized demonstration of the assigned technique and then proceeded directly to 5 consecutive recorded humeral head osteotomies on separate proximal humerus models using the same oscillating saw and blade type. Retroversion was assessed relative to the mount-based surrogate forearm axis in a manner analogous to intraoperative forearm referencing techniques commonly used during shoulder arthroplasty.

Pre-operative planning images demonstrating the target osteotomy orientation were displayed in the room to simulate standard pre-operative templating and intraoperative referencing. However, participants were only briefly allowed to visualize the models and planning images prior to osteotomy and were not permitted to extensively study the anatomy, rehearse the planned cut, or assess the accuracy of prior osteotomies. These measures were used to minimize iterative learning effects and reduce the possibility that repeated exposure to the model or planning images could artificially diminish differences between groups.

Three osteotomy attempts in the EG group were excluded from final analysis because loosening of the fixation apparatus resulted in non-planar cuts that could not be reliably reconstructed or analyzed using the software-based measurement methodology. These exclusions were related to fixation failure rather than osteotomy technique performance. Therefore, 42 osteotomies were included in the final analysis.

Following osteotomy, all models were digitized using a CR Scan 01 3D scanner to generate digital 3-dimensional reconstructions of each specimen. Entire digitized models were manually aligned and subsequently refined using the iterative closest point method within Blender version 4.3 (Amsterdam, Netherlands) (Fig. 4). The achieved osteotomy plane was manually identified and compared with the predefined reference osteotomy plane. Measurements were obtained for NSA, retroversion, and maximum cut height deviation. Primary outcomes included absolute deviation from the planned NSA, retroversion, and cut height. Retroversion deviation was calculated relative to the mount-based surrogate forearm axis. Measurements were reviewed by 2 reviewers, and final measurements were determined by a single reviewer who was not blinded to group assignment.

**Figure 4:**
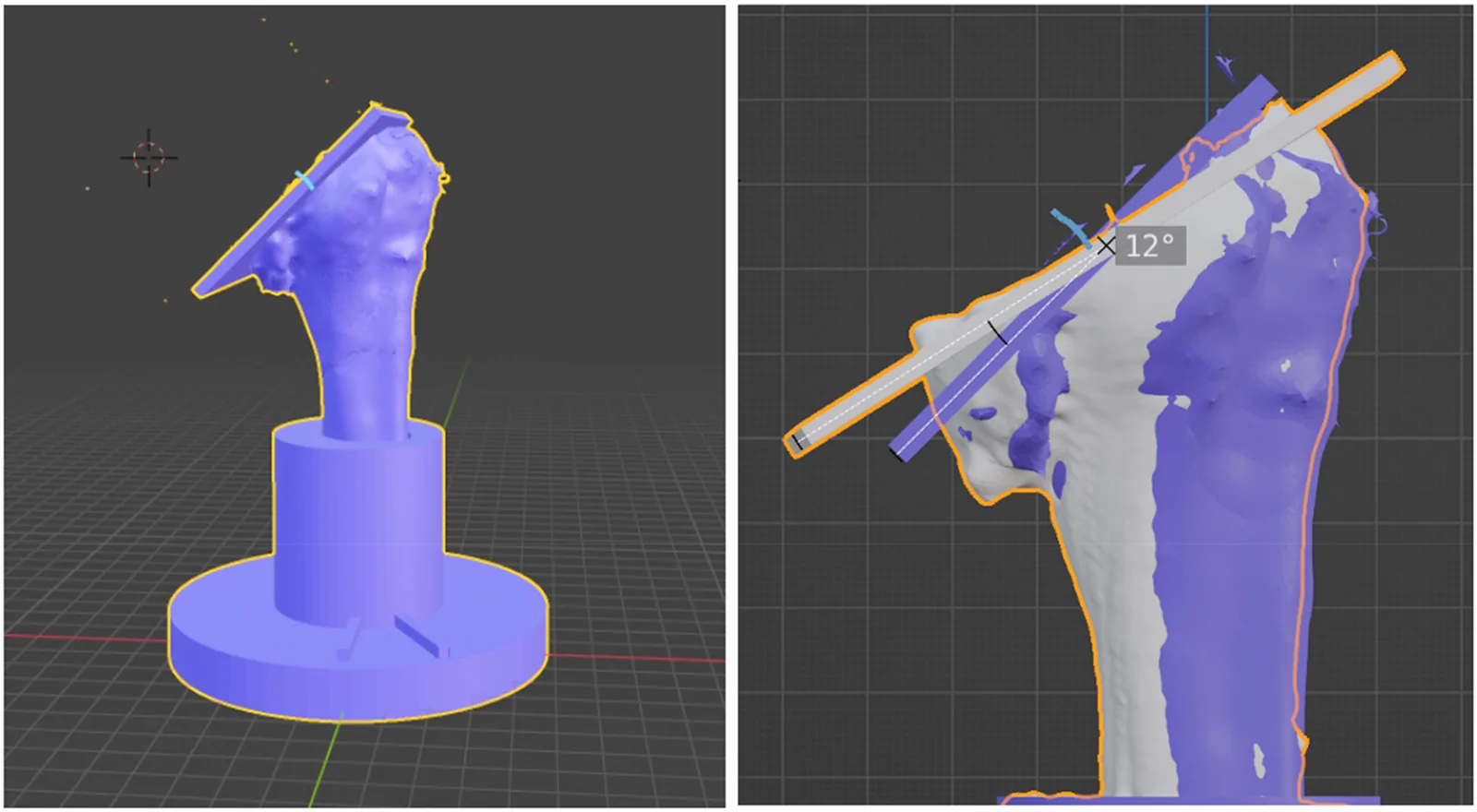
Three-dimensional software analysis and iterative closest point registration. Representative workflow demonstrating three-dimensional scanning, manual alignment, and iterative closest point (ICP) registration of osteotomy specimens to the predefined reference osteotomy model within Blender software for measurement of neck-shaft angle, retroversion, and cut height deviation.

Because each participant performed multiple osteotomies, linear mixed-effects models were utilized to account for clustering of repeated measurements within individual participants. The osteotomy technique was included as a fixed effect, and the participant was included as a random effect. Separate linear mixed-effects models were created for NSA deviation, retroversion deviation, and cut height deviation. Statistical significance was defined as *P* < .05. PGY was compared across technique groups using one-way analysis of variance. The association between PGY and osteotomy accuracy was assessed by Pearson correlation across the 9 participant-level mean absolute deviations, and PGY was also entered as a fixed-effect covariate in each mixed-effects model.

## Results

A total of 42 humeral head osteotomies were included in the final analysis, including 15 MR-assisted osteotomies, 15 freehand osteotomies, and 12 osteotomies performed using an EG (Table I). Three EG osteotomies were excluded because fixation loosening resulted in non-planar cuts that could not be reliably analyzed using the software-based measurement methodology.

Participant training level by group is shown in Table II. The mean training level was PGY-4.0 in the MR group and PGY-3.7 in both the freehand and EG groups and did not differ between groups (*P* = .932). Across the 9 participants, PGY was not associated with mean absolute deviation in NSA (r = 0.09, *P* = .809), retroversion (r = 0.35, *P* = .363), or cut height (r = 0.11, *P* = .774). Entering PGY as a covariate in each mixed-effects model did not attenuate the effect of technique on any outcome; point estimates for the difference between MR and each comparator were marginally larger after adjustment than before it.

**Table II:** Participant training level by technique group.

Technique group	Residents (n)	Postgraduate year of participants	Mean PGY
Mixed reality	3	PGY-3, PGY-4, PGY-5	4.0
Freehand	3	PGY-3, PGY-3, PGY-5	3.7
Extramedullary guide	3	PGY-2, PGY-4, PGY-5	3.7
All participants	9	PGY-2 to PGY-5	3.8

*PGY*, postgraduate year.

Postgraduate year (PGY) of the nine orthopedic surgery residents in each technique group. Training level did not differ between groups (one-way analysis of variance, *P* = .932). Across the nine participants, postgraduate year was not correlated with mean absolute deviation in neck-shaft angle (r = 0.09, *P* = .809), retroversion (r = 0.35, *P* = .363), or cut height (r = 0.11, *P* = .774). Prior experience performing a humeral head osteotomy during a case ranged from 0 to 10 osteotomies and correlated more with interest in shoulder arthroplasty than with training year.

MR-assisted osteotomy demonstrated significantly lower absolute NSA deviation compared with both freehand and EG techniques. Mean absolute NSA deviation was 5.6° ± 5.2° in the MR group, compared with 10.4° ± 6.3° in the freehand group and 10.4° ± 5.2° in the EG group. Linear mixed-effects modeling demonstrated significantly lower NSA deviation with MR guidance (*P* = .013).

MR guidance also demonstrated significantly lower absolute cut height deviation. Mean absolute cut height deviation was 4.2 ± 2.0 mm in the MR group, compared with 7.8 ± 3.0 mm in the freehand group and 7.1 ± 3.5 mm in the EG group. Mixed-effects analysis demonstrated significantly lower cut height deviation with MR guidance (*P* = .008).

No significant differences in retroversion deviation were identified between groups. Mean absolute retroversion deviation was 5.0° ± 3.3° in the MR group, 5.4° ± 3.9° in the freehand group, and 5.5° ± 3.1° in the EG group (*P* = .70).

Figure 5 demonstrates signed NSA and cut height deviation for each osteotomy attempt relative to the ideal target osteotomy. MR-assisted osteotomies demonstrated tighter clustering around the target center compared with freehand and EG techniques, indicating improved overall osteotomy precision and accuracy.

**Figure 5:**
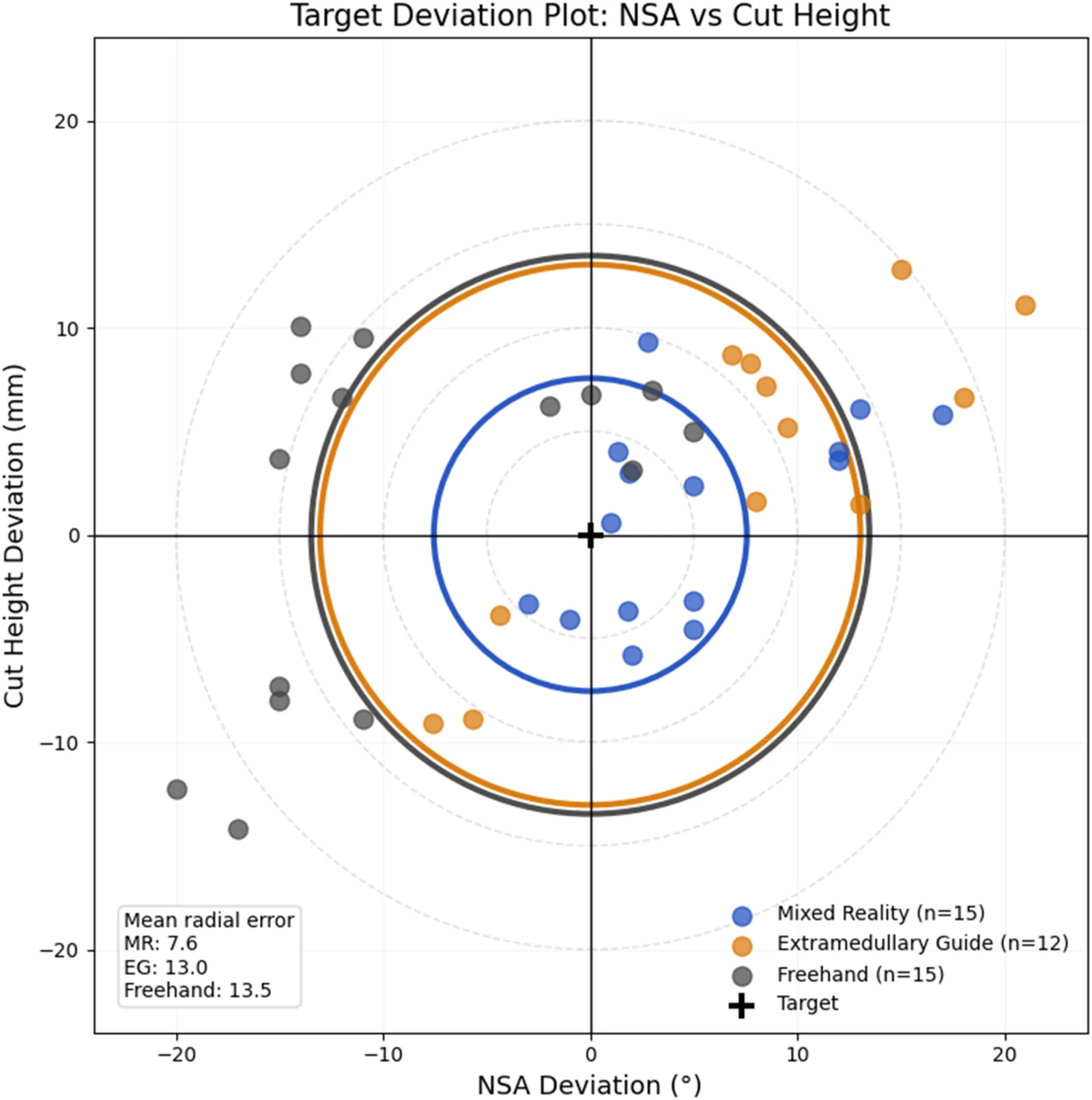
Target deviation plot demonstrating multidimensional osteotomy error. Target deviation plot demonstrating signed neck-shaft angle (NSA) and cut height deviation for each osteotomy attempt. The origin (0,0) represents the ideal planned osteotomy. Each point represents a single osteotomy attempt. Colored circles represent the mean radial error for each group, calculated as the Euclidean distance from the ideal target using combined NSA and cut height deviation. Mixed reality (MR)-assisted osteotomies demonstrated tighter clustering and lower overall multidimensional osteotomy error compared with freehand and extramedullary guide (EG) techniques.

## Discussion

The most important finding of this study was that MR holographic assistance improved the accuracy and precision of humeral head osteotomy NSA and cut height compared with both freehand and EG techniques. MR-assisted osteotomies demonstrated tighter clustering around the ideal osteotomy target on target deviation analysis, indicating improved reproducibility. In contrast, freehand and EG techniques demonstrated comparable accuracy for both NSA and cut height, while no significant differences in retroversion deviation were identified between groups.

Accurate humeral head osteotomy is critical in both anatomic and reverse shoulder arthroplasty because it directly influences implant alignment, soft tissue tensioning, joint stability, range of motion, and long-term implant survivorship. In anatomic shoulder arthroplasty, inaccurate restoration of inclination, version, or humeral height may result in altered glenohumeral biomechanics, eccentric glenoid loading, stiffness, instability, and accelerated component wear.^8^, ^9^, ^31^ In reverse shoulder arthroplasty, humeral osteotomy also affects deltoid tensioning, prosthetic stability, scapular impingement, arm lengthening, and acromial stress.^2^, ^14^, ^17^ Accordingly, improved osteotomy reproducibility may have important implications for implant positioning and shoulder biomechanics in both anatomic and reverse shoulder arthroplasty.

Freehand humeral osteotomy remains widely utilized because of its simplicity and efficiency but has demonstrated substantial variability in inclination, version, and cut height accuracy.^12^, ^29^, ^30^ EGs were developed to improve osteotomy reproducibility but remain dependent on surgeon technique and forearm referencing, which may introduce variability in version and inclination assessment.^16^ In the present study, EG-assisted osteotomy did not significantly improve accuracy compared with freehand osteotomy, suggesting that conventional extramedullary referencing alone may not sufficiently improve osteotomy reproducibility.

In the present study, the EG functioned as an unpinned physical reference rather than as a captured cutting guide, and no mark was made on any model. The osteotomy itself was therefore executed freehand in all 3 groups, and the comparison between MR and EG is a comparison between a holographic 3-dimensional reference and a physical reference to the same plane, with the cut performed identically in both. That MR guidance produced lower cut height deviation under these conditions suggests that the advantage lies in how the target plane is visualized.

Computer navigation, computed tomography-based planning, and patient-specific instrumentation have demonstrated improved implant positioning accuracy in shoulder arthroplasty but may increase cost, workflow complexity, and dependence on advanced imaging and pre-operative planning.^1^, ^4^, ^11^, ^22^, ^26^, ^32^ MR technology is an emerging alternative that may improve surgical visualization and spatial orientation while preserving intraoperative flexibility and workflow efficiency.

Prior studies evaluating MR guidance in shoulder arthroplasty have demonstrated improved glenoid guide pin positioning and greater spatial understanding during component placement.^6^, ^7^, ^10^, ^23^, ^28^ Erickson et al^6^ reported that MR holographic guidance improved glenoid guide pin placement accuracy in a B2 glenoid model and achieved accuracy comparable to patient-specific instrumentation while preserving intraoperative flexibility. Consistent with these findings, the present study demonstrated improved humeral osteotomy accuracy with MR guidance. These improvements were achieved without formal optical tracking, instrument tracking, or hologram registration. Rather than functioning as a true navigated platform, the MR system used in this study served solely as an interactive 3-dimensional holographic visual reference. The absence of formal registration limited true navigational capability but reduced workflow complexity and preserved intraoperative flexibility. Improved 3-dimensional visualization and spatial orientation alone may therefore improve humeral osteotomy reproducibility and reduce dependence on surgeon experience and subjective spatial estimation. Because the system required no navigation infrastructure, optical tracking, or patient-specific manufacturing, MR guidance may be a lower-workflow and more accessible adjunct than existing navigated technologies.

In the present cohort, PGY was balanced across the 3 technique groups and was not associated with osteotomy accuracy on any outcome, and adjustment for PGY did not attenuate the effect of technique. The differences observed between techniques are therefore unlikely to reflect differences in training level between groups.

Interestingly, retroversion deviation was not significantly improved with MR guidance. This likely reflects the inherent difficulty of assessing humeral version intraoperatively, particularly given variability in forearm referencing techniques and the absence of formal navigation or tracking within the current MR system. Unlike inclination and cut height, retroversion lacks a fixed visual reference within the exposed operative field and may therefore be less amenable to improvement through passive holographic visualization alone. Future MR systems incorporating registration, tracking, or instrument guidance may further improve version accuracy. The present study was also not specifically powered to detect small differences in retroversion deviation between groups.

Statistical improvement in osteotomy accuracy does not by itself establish clinical benefit. The magnitude of deviation in NSA, version, or cut height at which patient-reported outcomes, range of motion, or implant survivorship begin to suffer has not been defined for either anatomic or reverse shoulder arthroplasty. Biomechanical and computational studies demonstrate that inclination and height errors alter deltoid moment arm, joint reaction force, and impingement-free motion,^14^, ^17^, ^24^, ^31^ but these models do not identify a threshold beyond which an individual patient does worse. The differences observed in the present study fall within a range that biomechanical work suggests is mechanically meaningful, yet no clinical series has linked deviations of this magnitude to function, revision, or survivorship. Whether improved osteotomy accuracy translates into improved clinical outcomes can be answered only by clinical studies with long-term follow-up, and the present findings should be interpreted as a measure of technical accuracy rather than as evidence of clinical benefit.

This study has several limitations. Osteotomies were performed on Sawbones reproductions of a single arthritic proximal humerus, without surrounding soft tissue or an intact rotator cuff, which does not replicate the haptic feedback, exposure, or anatomic variability of live surgery and may understate intraoperative difficulty. The tradeoff is a controlled environment in which guidance technique was the only variable differing between groups. Participants performed 5 consecutive osteotomies, although planning time was limited and they could not review prior cuts. Version was assessed against a mount-based surrogate forearm axis, and the MR system functioned as passive holographic guidance rather than a navigated platform. The EG was designed and 3-dimensionally printed by the senior author and used unpinned as a physical reference, so the EG group represents extramedullary referencing rather than any commercial instrument. All participants were residents, and the training-level analysis of 9 participants was not powered to detect a modest association between PGY and accuracy; it establishes that training level was balanced across groups, not that training level is unrelated to osteotomy accuracy. Finally, the HoloLens 2 is no longer manufactured, and Microsoft has committed to software support only through 2027. The application was developed in Unity and is not specific to that headset, but replication on currently available head-mounted displays is necessary before these results generalize to systems now in use.

Future investigations should evaluate MR-assisted humeral osteotomy in cadaveric and clinical settings and determine whether improved osteotomy accuracy ultimately translates to improved implant positioning, biomechanics, and patient outcomes.

## Conclusion

This study demonstrated that MR holographic guidance improved the accuracy and reproducibility of humeral head osteotomy, particularly with respect to NSA and cut height, compared with both freehand and EG techniques. MR-assisted osteotomies demonstrated tighter clustering around the ideal target osteotomy, suggesting improved precision and consistency. Importantly, these improvements were achieved using passive holographic guidance without formal navigation, tracking, or hologram registration, suggesting that 3-dimensional visualization and spatial orientation alone may improve osteotomy performance. Future investigations should evaluate MR-assisted humeral osteotomy in cadaveric and clinical settings and determine whether improved osteotomy accuracy ultimately translates to improved implant positioning, biomechanics, and patient outcomes.

## Disclaimers:

Funding: No funding was disclosed by the authors.

Conflicts of interest: John Erickson reports that he and/or his immediate family have a financial interest in RSQ Holo, a mixed reality company, related to the subject of this article. Any additional authors, their immediate families, and any research foundations with which they are affiliated have not received any financial payments or other benefits from any commercial entity related to the subject of this article.

## Declaration of generative AI and AI-assisted technologies in the writing process

During the preparation of this work, the authors used ChatGPT (OpenAI, GPT-5.5) for language editing, grammar, formatting, and manuscript refinement. No AI-generated data, analysis, or figures were used. After using this tool, the authors reviewed and edited the content as needed and take full responsibility for the content of the published article.
